# New Real-Time High-Density Impulsive Noise Removal Method Applied to Medical Images

**DOI:** 10.3390/diagnostics13101709

**Published:** 2023-05-11

**Authors:** Turki M. Alanazi, Kamel Berriri, Mohammed Albekairi, Ahmed Ben Atitallah, Anis Sahbani, Khaled Kaaniche

**Affiliations:** 1 Department of Electrical Engineering, College of Engineering, Jouf University, Sakakah 72388, Saudi Arabia; 2 LAMMDA Laboratory, University of Sousse, Sousse 4054, Tunisia; 3 Institute for Intelligent Systems and Robotics (ISIR), CNRS, Sorbonne University, 75006 Paris, France

**Keywords:** medical images, high-density impulsive noise, image processing, high-level synthesis, FPGA

## Abstract

This paper introduces a new method for real-time high-density impulsive noise elimination applied to medical images. A double process aimed at the enhancement of local data composed of Nested Filtering followed by a Morphological Operation (NFMO) is proposed. The major problem with heavily noisy images is the lack of color information around corrupted pixels. We show that the classic replacement techniques all come up against this problem, resulting in average restoration quality. We only focus on the corrupt pixel replacement phase. For the detection itself, we use the Modified Laplacian Vector Median Filter (MLVMF). To perform pixel replacement, two-window nested filtering is suggested. All noise pixels in the neighborhood scanned by the first window are investigated using the second window. This investigation phase increases the amount of useful information within the first window. The remaining useful information that the second window failed to produce in the case of a very strong connex noise concentration is then estimated using a morphological operation of dilatation. To validate the proposed method, NFMO is first evaluated on the standard image Lena with a range of 10% to 90% impulsive noise. Using the Peak Signal-to-Noise Ratio metric (PSNR), the image denoising quality obtained is compared to the performance of a wide variety of existing approaches. Several noisy medical images are subjected to a second test. In this test, the computation time and image-restoring quality of NFMO are assessed using the PSNR and the Normalized Color Difference (NCD) criteria. Finally, an optimized design for a field-programmable gate array (FPGA) is suggested to implement the proposed method for real-time processing. The proposed solution performs excellent quality restoration for images with high-density impulsive noise. When the proposed NFMO is used on the standard Lena image with 90% impulsive noise, the PSNR reaches 29.99 dB. Under the same noise conditions, NFMO completely restores medical images in an average time of 23 milliseconds with an average PSNR of 31.62 dB and an average NCD of 0.10.

## 1. Introduction

Noise reduction is a critical process in image processing. It is necessary to reduce the level of noise present in an image to obtain clear, distinguishable shapes and structures. Achieving this requires understanding the sources of noise in the image and using various techniques to reduce these sources. Impulsive noise, also known as salt and pepper noise, is a type of noise with a high dynamic range that results from extreme pixel values. It is caused by errors in transmission, analog-to-digital conversion, and data acquisition. Impulsive noise can be quite challenging to reduce from an image, as it is typically randomly distributed, making it difficult to distinguish from the true image features. One strategy for reducing impulsive noise is using median filtering, which replaces each pixel with the median value of its neighbors [1,2,3,4,5]. This reduces the effect of impulsive noise while preserving other image features. Another option is to use a non-linear filter, such as a morphological filter [6,7,8,9]. This smooths the edges of objects while preserving their shapes and is effective at removing impulsive noise. Depending on the type of image to be processed, the amount of noise in the image, or the constraints on the execution time, several more sophisticated approaches have emerged. The weighted vector filters [10,11], adaptive vector filters [12,13,14,15,16,17,18,19], hybrid vector filters [20,21], fuzzy vector filters [22,23,24], and vector filters based on neural networks [25,26,27,28] are the most notable examples. 

Our objective in this study is to create a real-time architecture that can restore, as accurately as possible, medical images with high-density impulsive noise. The classification accuracy of these kinds of images is directly related to the quality of the restoration that is performed. Indeed, a variety of types of noise can distort medical images. Impulsive noise, Rician noise, Gaussian noise as well as quantum mottle noise are among the most common [29,30,31,32]. We are particularly interested here in impulsive noise. The classification of medical images has received increased interest from scientists over the past three decades [33]. In several clinical applications, medical images based on Magnetic Resonance (MRI), Computed Tomography (CT), or Positron Emission Tomography (PET) allow the diagnosis of pathologies, sometimes even very early. This allows the right plan for healing to be put in place [34]. 

Recent literature contains a multitude of methods for denoising images with high-density impulsive noise. There are first the methods employing the median filter. These methods are the most prevalent in published works. The goal is to improve the median filter’s ability to detect and restore corrupted pixels. Among these methods, we can specifically mention the Adaptive Switching Weighted Median Filter (ASWM) [35], the Noise Adaptive Fuzzy Switching Median Filter (NAFSM) [36], the Modified Decision-Based Un-symmetric Trimmed Median Filter (MDBUTMF) [37], and the Adaptive Dynamically Weighted Median Filter (ADWMF) [38]. There are also interpolation-based methods, such as the Adaptive Decision Based Inverse Distance Weighted Interpolation (DBIDWI) [39] and the Adaptive Decision based Kriging Interpolation Filter (ADKIF) [40]. A third family of methods exploits probability and statistical calculations to restore high-density impulsive noise images. Probabilistic Decision Based Filter (PDBF) [41], Adaptive Probability Filter (APF) [42], and Based-on-Pixel Density Filter (BPDF) [43] are distinguished members of this family. There are also proposals for methods that are less conventional, such as the Adaptive Content based Closer Proximity Pixel Replacement Algorithm (ACCPPRA) [44] based on a heuristic decision tree, the Adaptive Weighted Mean Filter (AWMF) [45] where the noise candidate is replaced by the weighted mean of a specific window, and the Adaptive Cardinal B-Spline Algorithm (ACBSA) [46] which perform a local cardinal B-spline proprieties analysis. All of the methods listed here aim to restore images with significant noise. They each exploit the neighborhood in their own way. This neighborhood is severely lacking in data, with the majority of pixels being corrupted. Even though these methods implicitly invoke an increase in useful information, none has explicitly proposed a thorough investigation of the neighborhood with the intention of restoring it before estimating the central corrupted pixel. 

In this paper, we propose a deep investigation of the area around corrupted pixels in order to obtain more useful information. Our algorithm performs a nested filtering process using two windows. The first, called the main window, will be centered around the noisy pixel. It will identify neighboring pixels in order to estimate its new value. These neighboring pixels are mainly noisy and do not participate in the estimation of the final value. To remedy this, a second window called the secondary window is launched around each corrupt neighboring pixel. The mission of the secondary window is to collect as much information as possible around each corrupt neighboring pixel and deduce its possible value from the median of this information. This process will increase the amount of non-noisy information around the main pixel to be corrected. Ideally, all noisy neighbors should be corrected. This is not always the case when the noise is very loud. At this moment, a morphological operation intervenes to complete the missing information. The nested filtering process leaves gaps that a proposed addition operation in the sense of Minkowski fills [47]. The proposed method is referred to as “NFMO” (Nested Filtering followed by a Morphological Operation) in the rest of this paper.

To evaluate the performance of the proposed NFMO, we proceed in two steps. Initial evaluation of NFMO is performed on the standard images Lena, Boat, and Baboon with 10% to 90% impulsive noise. Using the PSNR metric, the quality of images restoration is compared to the performances of ASWM, NAFSM, MDBUTMF, ADWMF, DBIDWI, ADKIF, PDBF, APF, BPDF, ACCPPRA, AWMF, and ACBSA in the case of Lena image. For Boat and Baboon images, the comparison using the PSNR metric is performed with MDBUTMF, DBIDWI, ADKIF, ACCPPRA, AWMF, and ACBSA. These methods were chosen for three reasons: They deal with the issue of denoising images highly contaminated by impulsive noise, are part of recent literature, and belong to a variety of families. A second, more specific evaluation of the NFMO’s performance is proposed in this work. In fact, in addition to the major contribution proposed by the NFMO, another substantial contribution is introduced. A hardware implementation of the NFMO is proposed. Our goal is to provide an embedded denoising algorithm that can be applied to medical images. As a result, a second evaluation is carried out on a set of high-density impulsive noisy medical images. The performance of the proposed NFMO is evaluated in terms of processing time and quality of image restoration as measured by the PSNR and the NCD metrics.

Hardware acceleration has been utilized by the scientific community to minimize implementation complexity. In [48], the authors employ two distinct hardware designs to create conventional and multi-level median filters. Ref.[49] presents a unique 3×3 window median filtering approach based on a bit-serial sorting algorithm with fast operating speed and minimal hardware complexity. In [50] is detailed a hardware implementation of the Vector Median-Rational Hybrid Filters for color images. Using approximations, this hardware design simplifies the implementation of relational functions. For effective Vector Median Filter implementation, ref.[51] suggests a fast parallel design. Vector Median Filter implementations in this design resemble L2 norms. Nevertheless, the creation of these hardware designs takes longer, and they cannot be modified. These systems are constructed and implemented using Low-Level Synthesis and Hardware Description Language (HDL) on a Field-Programmable Gate Array (FPGA) circuit. Using a Low-Level Synthesis design, the Register Transfer Level (RTL) description may be adjusted to generate an excellent and efficient netlist. Developing an RTL description is arduous and time-consuming, particularly for complicated applications [52,53,54]. In fact, each low-level circuit’s operations must be specified. Complex systems can only be developed by hardware designers with specific knowledge and abilities. In order to simplify the complexity of the FPGA design, Low-Level Synthesis must be replaced by High-Level Synthesis (HLS) [55,56,57]. Using software high-level languages (systemC, C/C + +, etc.), the HLS tool transforms coded algorithms into a structural and behavioral RTL hardware description. As a consequence, various commercial and academic HLS tools, such as Xilinx Vivado HLS, Intel OpenCL, Catapult-C, and ROCCC, are being developed. Thus, the goal of this research is to build several hardware designs for the NFMO’s proposed denoising algorithm utilizing HLS flow. When creating these designs, the cost and speed of the FPGA were considered. Using the Xilinx Zynq FPGA, the best-designed architecture will be built and evaluated.

This paper is organized as follows: In Section 2, MLVMF-based impulsive noise detection is presented. This algorithm will be used to detect corrupted pixels in the image. Proposed local data enhancement by nested filtering is the subject of Section 3, while Section 4 explains the missing information estimation process performed by the morphological tools. Section 5 shows the proposed NFMO performances using the standard images Lena, Boat, and Baboon, as well as a set of medical images. Finally, Section 6 describes the HLS designs for the proposed NFMO.

## 2. MLVMF-Based Impulsive Noise Detection

The impulsive noise removal process consists of two main steps: finding the noise and replacing the noisy pixel. Our first contribution in this work is to propose a method for replacing noisy pixels. For the detection phase, we are inspired by the very recent MLVMF method [58]. We have shown in [58] that the MLVMF has a high accuracy of impulsive noise detection, especially when the density of this noise is very high. In addition, the MLVMF has been designed for a hardware implementation, which fits perfectly with our final objective: to propose an embedded algorithm for the denoising of highly corrupted medical images.

**Figure 1:**
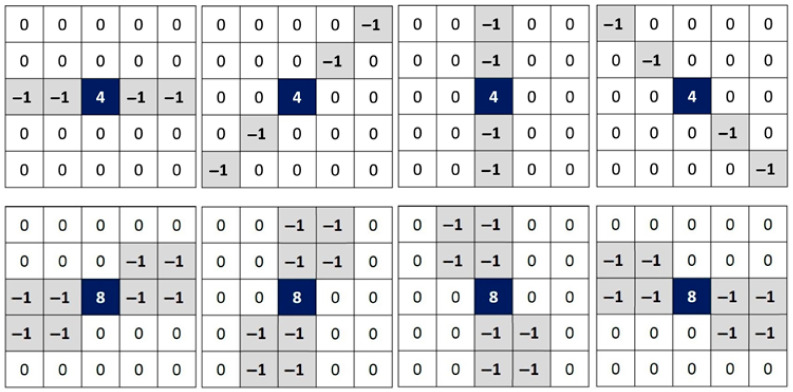
The first set of filters used by the MLVMF to identify corrupted pixels [58]. Eight kernels are proposed. From left to right from top to bottom: $K_{1}$ at 0, $K_{2}$ at $\frac{\pi }{4}$, $K_{3}$ at $\frac{\pi }{2}$, $K_{4}$ at $\frac{3\pi }{4}$, $K_{5}$ at $\frac{\pi }{8}$, $K_{6}$ at $\frac{3\pi }{8}$, $K_{7}$ at $\frac{5\pi }{8}$, and $K_{8}$ at $\frac{7\pi }{8}$.

**Figure 2:**
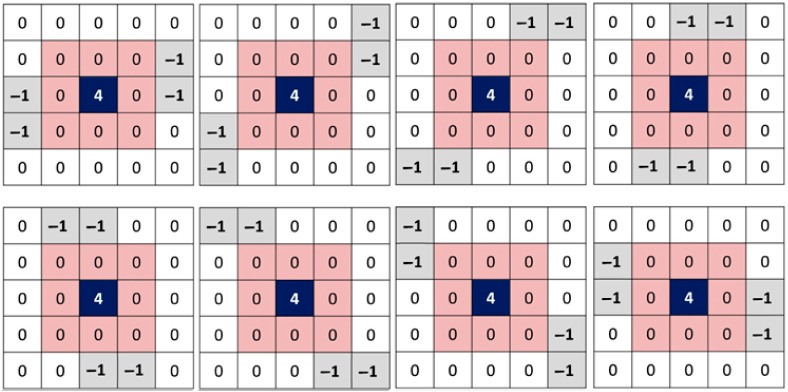
The second set of filters used by the MLVMF to identify corrupted pixels [58]. Eight more kernels are proposed. From left to right from top to bottom: $K_{9}$ to $K_{16}$.

MLVMF uses a modified Laplacian filter with a rotation step of π/8 to observe the intensity variations around each pixel of an image. Classic Laplacian filters typically employ rotation steps of π/2 or π/4. Reducing the angle of rotation around the pixel enables the use of additional information about its surrounding area. Figure 1 shows the first set of filters used by the MLVMF to identify corrupted pixels. However, the identification process can still fail in the presence of a significant amount of impulsive noise. Two or more pixels with identical intensities (0 or 255) can be 4-connected or 8-connected if they are noisy. The local second derivative surrounding this type of pixel will be incapable of detecting significant variations. A second round of searching is initiated to identify the neighboring noisy pixels. A second set of filters, described in Figure 2, calculates, in eight directions, the intensity variations surrounding each pixel of the image, ignoring the eight pixels that are immediately adjacent.

For each position (x,y) in the color image I(n×m×3), MLVMF compute the absolute value of the convolution product noted $V_{ij}$ between the kernel $K_{i}$ and the image $I_{j}$.

(1) $$V_{ij}(x,y)=\left|K_{i}⊗I_{j}\right|$$

where j varies from 1 to 3, such as $I_{1}$ is the image red component, $I_{2}$ is image green component, and $I_{3}$ is the image blue component. The scan of the image is performed in the first round with filters $K_{1}$ to $K_{8}$, and in the second round with filters $K_{9}$ to $K_{16}$. For each image pixel, we, therefore, obtain 24 measurements of variation by round. To judge the importance of the intensity variation around a pixel (x,y), we proceed as follows (Algorithm 1):
**Algorithm 1.** Thresholding process.${M_{1}\left(x,y\right)=\min[V}_{ij}\left(x,y\right),i=1..8,j=1..3]$ *if* $M_{1}\left(x,y\right)>T$ *% T is a predefined threshold*  *then* 
$\left(i_{ind},j_{ind}\right)=\mathrm{index}({\min[V}_{ij}\left(x,y\right),i=1..8,j=1..3])$  ***else***      ${M_{2}\left(x,y\right)=min[V}_{ij}\left(x,y\right),i=9..16,j=1..3]$     ***if*** $M_{2}\left(x,y\right)>T$       ***then*** $\left(i_{ind},j_{ind}\right)=\mathrm{index}({\min[V}_{ij}\left(x,y\right),i=9..16,j=1..3])$     ***end*** ***end*** *if* $I_{j_{ind}}\left(x,y\right)=\;0\;\mathrm{or}\;255$  *then* $\mathrm{pixel}\left(x,y\right)\mathrm{is}\;\mathrm{an}\;\mathrm{impulsive}\;\mathrm{noise}$ ***end*** 

## 3. Local Data Enhancement by Nested Filtering

Once the MLVMF has detected all of the pixels corrupted by impulsive noise, two windows of sizes sw1 and sw2 are created. The process of increasing information around each noise pixel is as follows: Centered at each noise pixel p(x,y), the main window of size sw1 will identify the neighborhood of this pixel. For each noise pixel $pn\left(i,j\right)\{i\neq x;j\neq y\}$ in this neighborhood, the secondary window (the nested window) of size sw2 centered at pn(i,j) will be used to search for all the non-noisy pixels. The noisy pixel pn(i,j) is replaced by the median value of the non-noisy pixels found by the secondary window if it exists. When the secondary window is done looking at the whole area outlined by the main window, two options become clear: the first option occurs when the neighborhood defined by the main window is completely restored. Typically, this occurs when the impulsive noise present in the image is of low density. The second option takes place when part of the neighborhood defined by the main window is restored while the rest of the corrupted neighborhood keeps its noisy values. This option is more common when the impulsive noise present in the image is of high density. Edge handling is performed using the mirror technique. Mirror size Ms is defined by: $Ms=Int\left(\frac{sw1}{2}\right)+Int\left(\frac{sw2}{2}\right)$.

**Figure 3:**
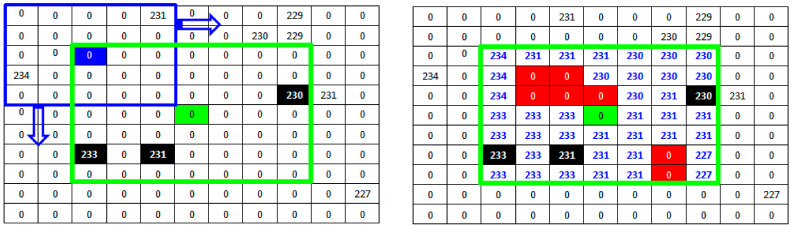
Double filtering process applied on a part of the 512 × 512 Lena standard image with 90% noise. The main filter (window) is in green. The nested (secondary) filter (window) is in blue.

Figure 3 shows an example of how nested filters can add to the information when the density of impulse noise is 90%. In Figure 3, the double filtering process is used on a part of the 512×512 Lena standard image (red layer) that has 90% noise. Corrupted pixels are assigned the value zero. The main filter of size sw1=7 (in green) is centered on the noisy pixel with coordinates x=100,y=40 (also in green). Three useful values (in black) among the 48 scanned by this filter are available. The rest of the pixels are noisy. It is the role of the secondary filter of size sw2=5 (in blue) to position itself around these noisy pixels in order to estimate their respective values if possible. 38 of the 45 noisy pixels were restored thanks to this operation (new values in blue). The remaining 7 pixels keep their noisy values (in red). The impulsive noise around these 7 pixels is so dense that no useful value appears in the neighborhood created by the nested (secondary) filter. Several experimental tests were carried out to measure the effect of the choice of sizes sw1 and sw2 of the main and secondary windows. For an image of size 512×512, the best restoration results are given by sw1=7 and sw2=5. It is true that increasing the size of these two windows can bring back more information, but at the risk of accentuating the redundancy. Thus, a smoothing phenomenon may arise and bias the quality of the final image by eliminating important details. On the other hand, if there is a lot of noise around a pixel and the size of the windows is decreased, the useful information around the pixel will be reconstructed at a very slow rate. 

## 4. Missing Information Estimation by Morphological Operations

**Figure 4:**
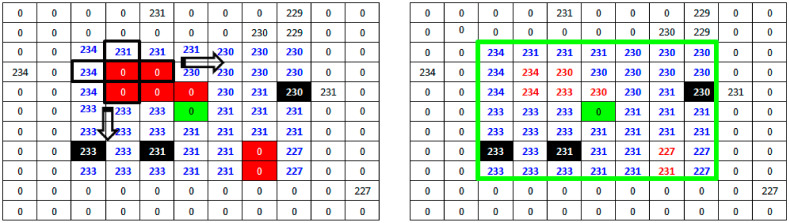
Dilatation operation applied on a part of the 512 × 512 Lena standard image with 90% noise. The main filter (window) is in green. The structuring element B is in black.

As mentioned in Section 3, if there is a lot of noise in an image, the process of adding more useful information around each noise pixel may not be enough. An additional operation is required for nested filtering. If we consider the set of pixels provided by the secondary window scan in addition to the already existing non-noisy pixels as an “object” set, and if we consider the remainder of the noise-corrupted pixels as a “non-object” set, then the binary configuration for the morphological operators is very appropriate. Let us consider B as a 4-connex structuring element:

(2) $$B=\left(\begin{array}{ccc} 0 & 1 & 0\\ 1 & ⟦1⟧ & 1\\ 0 & 1 & 0 \end{array}\right),$$

where ⟦1⟧ the origin of B. For each remaining noise pixel of the nested filtering phase, we propose a particular dilatation operation in order to estimate a new value of this pixel. Let X denote the set of pixels provided by the secondary window scan in addition to the already existing non-noisy pixels. Let E denote the space including all pixels inside the main window. Proposed dilatation process starts when the structuring element B, located by its origin, is moved on all positions of the space E. For each position corresponding to a non-restored pixel $p_{E}$, we check if B intersect X. The non-noisy pixels belonging to this intersection are candidates to replace the non-restored pixel $p_{E}$. To avoid information redundancy during this dilatation phase, the choice of the replacing pixel will be made by alternating the maximum and minimum of the candidate values. Figure 4 shows an example of the proposed dilatation operation. Based on the result provided by the nested filtering processing of Figure 3, the structuring element B (in black) is positioned on the first non-restituted pixel of the previous phase. Two useful values are detected: 231 and 234. Starting the alternation with the maximum, the new value of the pixel will be estimated at 234. The process continues from left to right and from top to bottom for all the noisy pixels until scanning the entire main window (in green). Figure 4 also shows that inside the main window (in green), centered on the noisy pixel (also in green), all the information has been reconstructed, first by nested filtering and then by the morphological operation of dilatation. Initially, only three useful values among the 48 possible values in the 7×7 neighborhood of the corrupted pixel are available to replace it. The proposed approach succeeds after a deep investigation of the neighborhood to provide an estimate of the remaining 45 values. It is now enough to replace the noisy pixel with the median value of its new neighborhood. In the example of Figure 4, the new value of the noisy pixel will be 231.

## 5. NFMO Performances

### 5.1. NFMO Performances Evaluation Using the Standard Lena Image

Performance evaluation denoising is typically done using the PSNR metric. By comparing the original image to the denoised image and measuring the amount of distortion, the PSNR provides a measure of the quality of the denoising process. For further analysis, the PSNR of a denoised image should typically be between 22 and 24 dB, with higher values indicating better denoising performance. As a result, it is an excellent metric for assessing the performance of denoising algorithms. Equations (3)–(5) define the PSNR:

(3) $$PSNR=10\log\left(\frac{255^{2}}{MSE}\right),$$

(4) $${MSE}_{l}=\frac{1}{nm}\sum_{i=1}^{n}\sum_{j=1}^{m}{\left(I_{l}\left(i,j\right)-I_{cl}(i,j)\right)}^{2},$$

(5) $$MSE=\frac{{MSE}_{r}+{MSE}_{g}+{MSE}_{b}}{3},$$

where l is the color channel index of a color image (r=red,g=green,b=blue). The initial image is denoted by I. The filtered image is denoted by $I_{c}$. n is the number of rows and m is the number of columns in an image. First, a PSNR comparison is performed between our proposed NFMO and the MLVMF. The MLVMF is based on the detection of noisy pixels by investigating the surroundings of each pixel of the image thanks to the 16 Laplacian filters presented in Figure 1 and Figure 2. Once noisy pixels are detected, the median is used to replace each of these pixels. The proposed NFMO uses the same approach to detect noisy pixels. Then, two nested filters and a dilation operation are used to estimate all pixels in the neighborhood and derive the replacement value. Table 1 shows PSNR values for both MLVMF and NFMO applied to the 512×512 Lena standard image corrupted with different impulsive noise densities (from 10% to 90%). We notice, thanks to Table 1, that the two methods give very close PSNR values up to a noise density equal to 30%. Beyond this value, the proposed NFMO becomes more and more efficient as the noise density increases. This result validates our proposition. The increase in useful information around noisy pixels has more impact when the neighborhood is poor (i.e., when the noise density is high).

**Figure 5:**
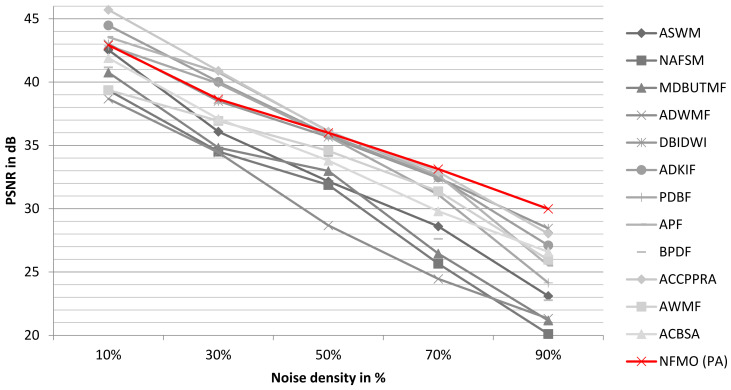
The variation of the PSNR according to the impulsive noise density injected into the Lena image. In grayscale, the PSNR variations of the 12 selected methods. The PSNR variation for the proposed NFMO method is shown in red.

**Figure 6:**
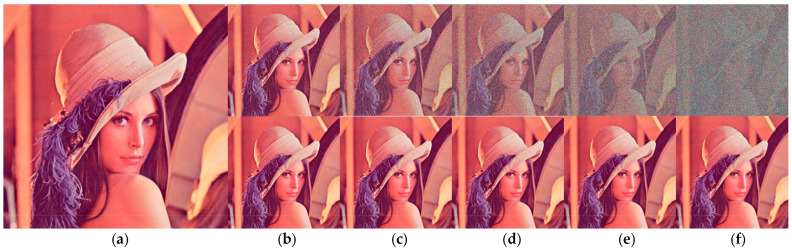
Qualitative evaluation of the NFMO performances applied to the Lena 512×512 color image. (**a**) Original Lena image. Top (**b**–**f**) Noised Lena images with noise density 10%, 30%, 50%, 70%, and 90%. Down (**b**–**f**) Filtered Lena Images by NFMO.

The quality of the proposed NFMO image restoration is now compared to the performances of ASWM, NAFSM, MDBUTMF, ADWMF, DBIDWI, ADKIF, PDBF, APF, BPDF, ACCPPRA, AWMF, and ACBSA using the PSNR metric. Three factors influenced the selection of these methods: They deal with the problem of denoising images that have been heavily contaminated by impulsive noise, they are recent, and they belong to a variety of families. In Table 2, the chosen methods are listed, along with a description of how they work. The Lena standard image is used in this step. Table 3 shows the PSNR performances of selected methods as well as the performances of the proposed NFMO method at different noise densities (from 10% to 90%) on the Lena standard image. Figure 5 illustrates the PSNR variation as a function of the impulsive noise density injected into the Lena image. The PSNR variations of the 12 selected methods are presented in grayscale. The proposed NFMO method’s PSNR variation is displayed in red. The proposed method works, as shown by the results in Table 3 and Figure 5. Beyond 50% impulsive noise, the NFMO gives the best PSNR and, therefore, the best denoising quality of all the methods. The local data enhancement proposed in the case of an image too poor in useful pixels succeeds in providing the best estimate of the corrupted pixels. Additionally, the deep investigation that the NFMO performs makes more sense when the noise is very loud. Indeed, below 50% of the impulsive noise injected, the NFMO gives a very good result without being exceptional. The lower the noise, the more useful information is available. The increase in information, in this case, loses its meaning. Figure 6 shows a visual qualitative evaluation of the performance of the NFMO applied to the Lena 512 × 512 color image with impulsive noise of density 10%, 30%, 50%, 70%, and 90%. We can clearly notice the very good reconstruction of the image with all its details.

**Figure 7:**
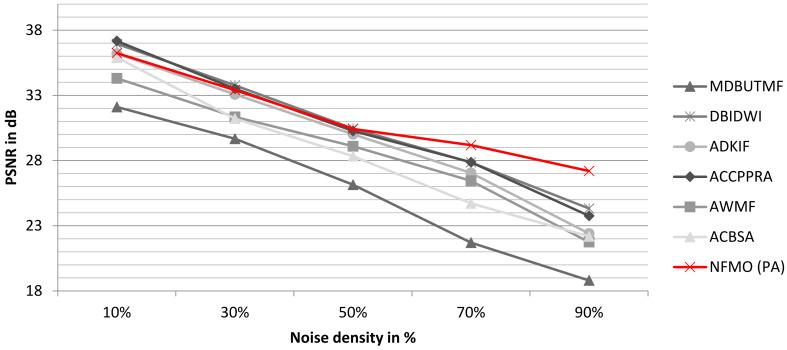
The variation of the PSNR according to the impulsive noise density injected into the Boat image. In grayscale, the PSNR variations of the selected methods. The PSNR variation for the proposed NFMO method is shown in red.

**Figure 8:**
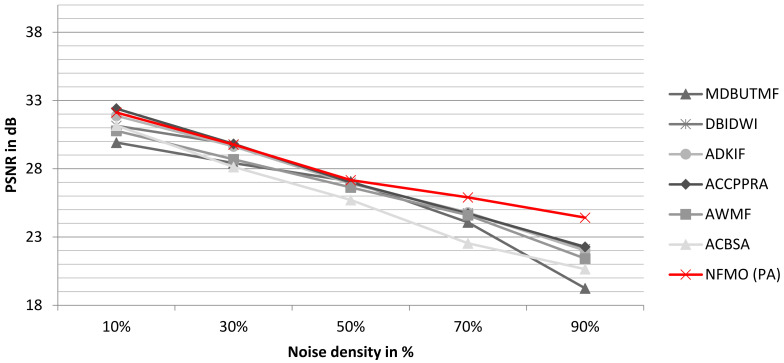
The variation of the PSNR according to the impulsive noise density injected into the Baboon image. In grayscale, the PSNR variations of the selected methods. The PSNR variation for the proposed NFMO method is shown in red.

**Figure 9:**
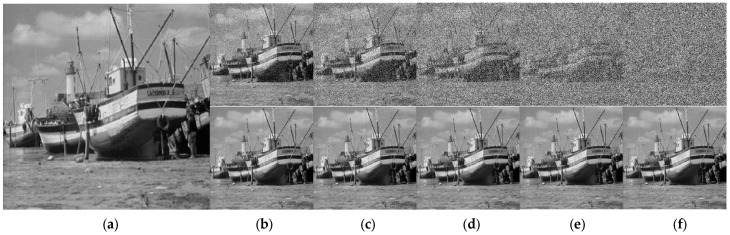
Qualitative evaluation of the NFMO performances applied to the Boat 512×512 image. (**a**) Original Boat image. Top (**b**–**f**) Noised Boat images with noise density 10%, 30%, 50%, 70%, and 90%. Down (**b**–**f**) Filtered Boat Images by NFMO.

**Figure 10:**
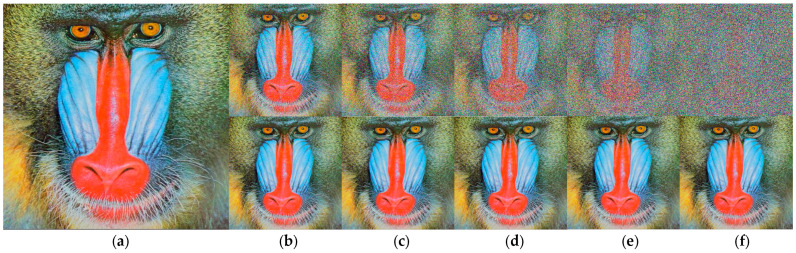
Qualitative evaluation of the NFMO performances applied to the Baboon 512×512 color image. (**a**) Original Baboon image. Top (**b**–**f**) Noised Baboon images with noise density 10%, 30%, 50%, 70%, and 90%. Down (**b**–**f**) Filtered Baboon Images by NFMO.

To better observe the performance of the proposed algorithm, we performed two additional tests on the standard images “Boat” and “Baboon”. We compare the PSNR performance of our proposed NFMO with the MDBUTMF, DBIDWI, ADKIF, ACCPPRA, AWMF, and ACBSA methods. Table 4 shows the PSNR performances of these methods as well as the PSNR performances of the proposed NFMO method at different noise densities (from 10% to 90%) on the Boat standard image. Table 5 shows the same comparison study performed on the Baboon standard image. Figure 7 and Figure 8 illustrate the PSNR variation as a function of the impulsive noise density injected, respectively, into the Boat and Baboon images. The PSNR variations of the six selected methods are presented in grayscale. The proposed NFMO method’s PSNR variation is displayed in red. Figure 9 and Figure 10 show a visual qualitative evaluation of the performance of the NFMO applied, respectively, to the Boat and Baboon 512 × 512 images with impulsive noise of density 10%, 30%, 50%, 70%, and 90%. These two test sets confirm the results obtained on the Lena image. The NFMO is efficient in the case of a low noise density and becomes more and more efficient when the noise density increases, giving the best values of PSNR among the other methods. It is, however, necessary to specify that a statistical validation of the proposed method to confirm the very satisfactory results found would be important. This requires further statistical study using a dataset of at least 100 images.

### 5.2. NFMO Performances Evaluation with Medical Images

The objective of this work is to provide a real-time filtering tool for medical images presenting strong impulsive noise. We have proposed a new method for replacing noisy pixels capable of restoring highly noisy images with very good quality. We are interested here in the quantitative and qualitative evaluation of the performance of the NFMO algorithm applied to a set of medical images. Medical images are selected from the Retinal Fundus Multi-Disease Image Dataset (RFMiD) 2.0 [59], the Alzheimer’s Dataset available in the Kaggle platform [60], and the COVID-19 Image Repository [61]. Six images were chosen to perform the experimental tests. CI1 and CI2 consist of brain MRI images with moderate dementia. CI3 and CI4 show color retina fundus: CI3 shows an anterior ischemic optic neuropathy, while CI4 shows coloboma in the macula and optic disc. CI5 and CI6 are chest x-ray images presenting, respectively, a moderate case and an advanced case of COVID-19. All these images have been normalized to a size of 256×256. Figure 7 shows the selected images.

**Figure 11:**
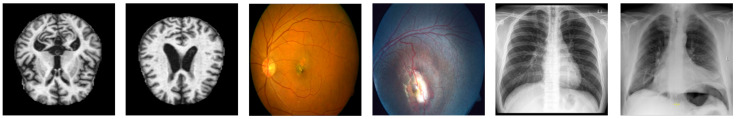
Medical images selected from the Retinal Fundus Multi-Disease Image Dataset (RFMiD) 2.0, Alzheimer’s Dataset available in the Kaggle platform, and COVID-19 Image Repository. From left to right: CI1, CI2, CI3, CI4, CI5, and CI6.

**Figure 12:**
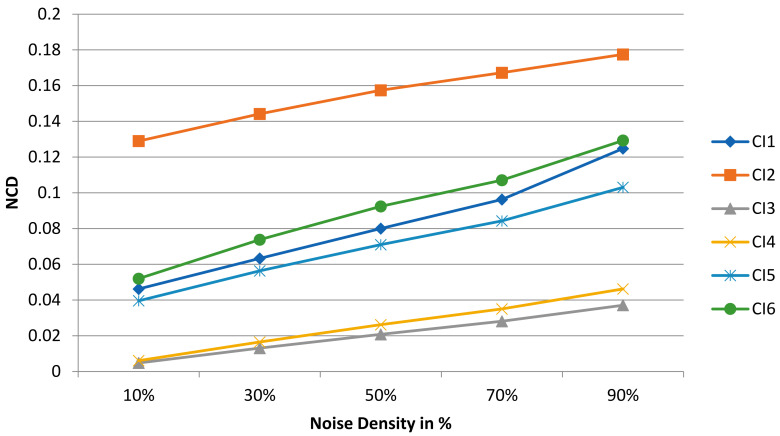
The evolution of the NCD when the impulsive noise density increases for CI1, CI2, CI3, CI4, CI5, and CI6.

**Figure 13:**
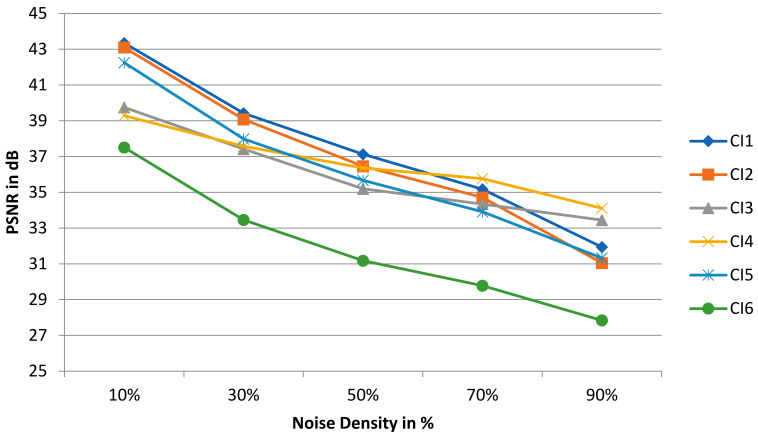
The evolution of the PSNR when the impulsive noise density increases for CI1, CI2, CI3, CI4, CI5, and CI6.

**Figure 14:**
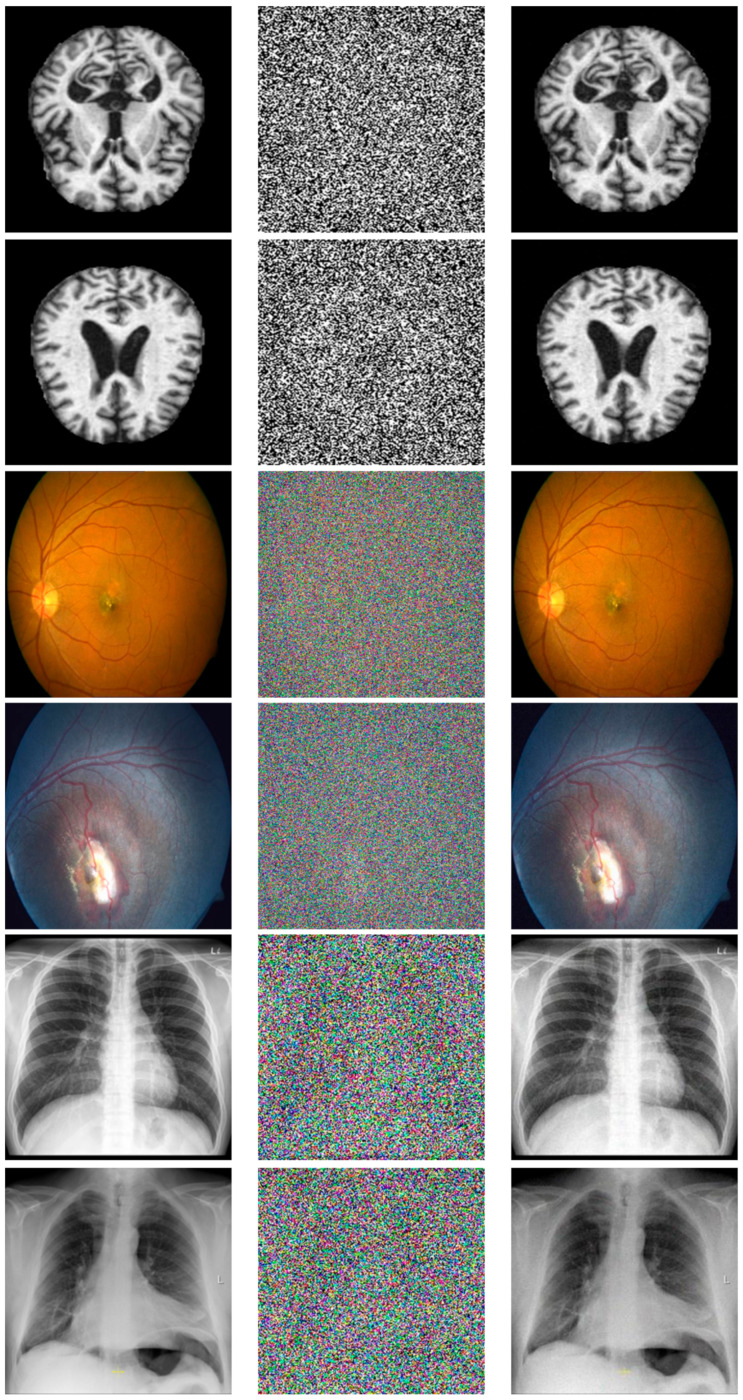
Qualitative evaluation of the NFMO performances applied to the selected medical images. First column: Original images Middle column: Original images corrupted by impulsive noise at 90%. Last column: Filtered images.

The performance evaluation of the NFMO on the selected medical images is carried out using two measures: the PSNR, which provides a measure of the quality of the denoising process, and the NCD (Normalized Color Difference), which provides a measure of the color distortion caused by the pixel replacement process. The value of the NCD is inversely proportional to the quality of restoration. The closer the colors in the filtered image are to the colors of the original image, the lower the NCD. NCD is calculated as follows:

(6) $$NCD=\frac{\sum_{i=1}^{n}\sum_{j=1}^{m}\sqrt{{\left(Y\left(i,j\right)-Y_{c}(i,j)\right)}^{2}+{\left(U\left(i,j\right)-U_{c}(i,j)\right)}^{2}+{\left(V\left(i,j\right)-V_{c}(i,j)\right)}^{2}}}{\sum_{i=1}^{n}\sum_{j=1}^{m}\sqrt{{\left(Y\left(i,j\right)\right)}^{2}+{\left(U\left(i,j\right)\right)}^{2}+{\left(V\left(i,j\right)\right)}^{2}}},$$

where Y is luminance of the original image, $Y_{c}$ is the luminance of the filtered image, U and V are the chrominance components of the original image, $U_{c}$ and $V_{c}$ are the chrominance components of the filtered image. Figure 11 also shows that the selected medical images can be color images or grayscale images. This does not affect our algorithm. Indeed, we have defined as the input of the proposed code a color image, which is therefore presented in the form of three layers: R, G, and B. A test is carried out during the reading of the image. If the image has a single layer, then it is a grayscale image. The algorithm then adds two other similar layers, and the noising and denoising processes can then begin. By adding these two layers, the image remains the same in terms of visual appearance, i.e., a grayscale image, but the noise can still attack any pixel of any layer. As for the NCD metric, it still remains valid since it requires three layers, which is the case thanks to the test phase explained above. The NCD provides a measure of the color distortion caused by the pixel replacement process, which remains valid even in the case of a grayscale image as long as it is presented in three layers and the noise affects these three layers independently. Table 6 shows measurements of the PSNR and NCD for the six medical images with different amounts of impulsive noise (from 10% to 90%). Figure 12 and Figure 13 reproduce the results of Table 6, highlighting the evolution of the PSNR and NCD measurements when the density of the impulsive noise increases. Figure 14 shows a visual qualitative evaluation of the performance of the NFMO applied to the selected medical images with impulsive noise of density equal to 90%.

The filtering operation by the proposed approach on medical images succeeded in restoring the original visual information even in the presence of very high-density impulsive noise. The CI3 and CI4 images present the best results in terms of denoising quality (high PSNR) and color fidelity (low NCD). This result makes perfect sense given the low frequency of repeating patterns in CI3 and CI4 compared to the rest of the test images. However, even though the CI1, CI2, CI5, and CI6 images all have a dominant texture, the measured PSNRs are still high, and the color distortion (NCD) is low. The visual evaluation presented in Figure 14 confirms the measurements in Table 6. We can note the great similarity between the original images and the filtered images, even in the absence of 90% of useful pixels. This is precisely the point of the local data enhancement offered by the NFMO. The average execution time of the proposed NFMO under Core™i7–1165g7@2.80ghz Intel processor reaches 471 sec per 256 × 256 color image when corrupted by 90% density noise. Deep investigation based on nested double filtering is indeed very costly in terms of computation time. Additionally, this calculation time is proportional to the density of the injected noise since the number of pixels to be corrected is greater when the density of the injected noise is high. Section 6 describes a proposed High-Level Synthesis (HLS) architecture for the NFMO filter to reduce execution time and enable real-time computation.

## 6. NFMO Real-Time Implementation

**Figure 15:**
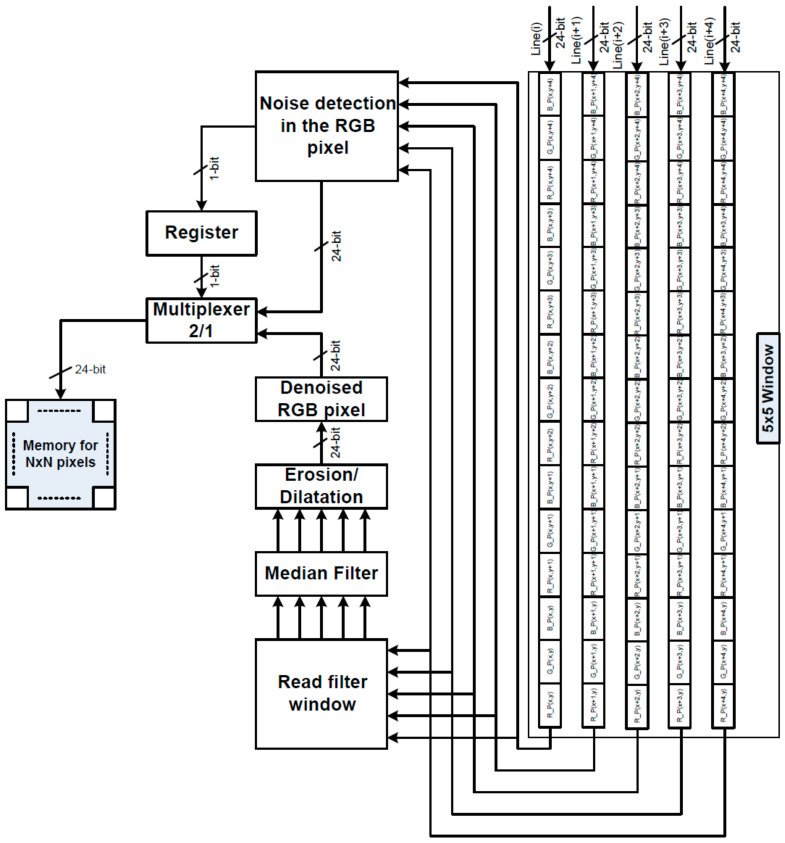
Proposed NFMO filter block diagram.

When combined with FPGA, the HLS process gives engineers a useful tool for quickly exploring the design space based on a high-level programming language (such as SystemC or C/C++) that describes how the system works. So, the HLS has turned into a useful and effective tool that could increase the amount of work that can be done during the design process and shorten the time it takes to finish a design cycle. In this situation, a number of HLS tools have been made, such as the Xilinx Vivado HLS tool, which gives a number of steps or directives for making the best hardware design for every algorithm. In fact, the RESOURCE directive makes it possible to construct arrays either as registers or as memory. In addition to this, the ALLOCATION directive may be used to improve the efficiency with which the arithmetic operation is performed. In addition to this, the ALLOCATION directive may be used to improve the efficiency with which the arithmetic operation is performed. Additionally, the loops can be pipelined, not unrolled, or fully/partially unrolled using the PIPELINE and UNROLL directives to improve the speed of loop iterations (i.e., to reach a higher throughput). Figure 15 displays the block diagram of the NFMO filter’s built hardware architecture. From a specified C/C + + code, the Xilinx Vivado HLS 18.1 tool performs the generating process. In order to boost the throughput of our NFMO filter, our architecture employs 5 DMA (Direct Memory Access) to transmit 5 images lines in parallel. The suggested NFMO’s hardware design is composed of 7×7 and 5×5 nested filters. Each denoised 24-bit RGB pixel is concatenated and stored in 256×256×24-bit internal memory. Certain directives (such as PARTITION and PIPELINE) are progressively added to the NFMO C/C++ code to improve the architecture. Thus, we build numerous NFMO hardware designs. Then, we preserve the optimal design, which strikes a balance between hardware cost and processing time. The hardware requirements for the various NFMO designs on the Zynq XCZU9EG FPGA in terms of Lookup-Table (LUT), Flip-Flops (FF), BRAM blocks, and DSP blocks are presented in Table 7.

Table 7 displays Solution 1’s hardware design without optimization. It is evident that this technique is not resource-hungry, but filtering the entire image needs a huge number of clock cycles. Solution 1 requires 1913 milliseconds for 256×256 color image with a clock frequency of 115 MHz and 90% density noise. We propose to implement the PIPELINE directive in Solution 2. The cost of hardware grows yet remains significantly below the system’s maximum capacity. In contrast, the number of clock cycles drops by 97.5%. Hence, an image may be processed in 48 milliseconds. In order to further reduce the number of clock cycles, Solution 3 includes the PARTITION directive, which allows the filtering window (Figure 15) to be divided into tiny blocks and promotes parallel data access. Solution 3 achieves a computation time of 23 milliseconds, which is 83 times quicker than Solution 1. More hardware resources will be required, yet hardware capacity will not be exceeded. Lastly, it should be noted that the software implementation of Section 5 yielded the same filtering quality with both Solution 2 and Solution 3.

## 7. Conclusions

In this work, a real-time tool for the elimination of high-density impulsive noise is proposed. Applied to heavily corrupted medical images, the proposed algorithm achieves excellent restoration quality. Two major contributions are at the center of this paper. The first consists of a new algorithm for replacing noisy pixels. This algorithm increases the useful local information to allow a better estimation of the pixel. This operation is based on double-nested filtering followed by a morphological operation of dilation, hence the name of this new method: the NFMO. Nested filtering is very efficient. The NFMO achieves the best performance in terms of denoising quality (PSNR) among the most recent and best-known methods in the literature when impulsive noise density exceeds 50%. Applied to medical images, NFMO restores color information with excellent PSNR and NCD metrics. These images are characterized by the presence of repetitive patterns and, in some cases, a predominantly black background. Visual information is, therefore, not very diversified. The results obtained in this paper show that the performance of the NFMO increases with the density of the impulsive noise injected while also being very good when this density is low. This is a very original result, opening perspectives on the uses of the NFMO in several other fields in addition to medical imaging. The very good quality of denoising obtained by the NFMO has, however, a cost. The nested nature of the proposed method required quite a lot of computational time. The second contribution in this paper solves this problem. A hardware architecture is proposed, allowing the filtering of corrupted 256×256 images with 90% impulsive noise in an average of 23 milliseconds. When integrated into the medical image acquisition chain, the proposed tool can guarantee excellent filtered image quality, allowing for better manual or artificial intelligence-based diagnosis.

In this work, we are interested in impulsive noise that can bias medical images. However, other types of noise can also attack these images. In particular, the Rician noise, which is very common in MRI images, or even the quantum mottle noise, which attacks CT and X-ray images. How will our proposed NFMO behave in front of these types of noises? If the reconstitution of the signal by the NFMO must logically work since it is based on useful existing information, the detection by the modified Laplacian filters of Rician-type noises or even quantum mottle noise remains to be validated. It is quite possible that slight or deep modifications must be made to the detection phase. Future work will focus on deepening the investigation into this issue.

## Figures and Tables

**Table 1:** Performances of the MLVMF and performances of the proposed NFMO method at different noise densities (from 10% to 90%) for PSNR on the Lena standard image.

Noise in % 🡪	10%	30%	50%	70%	90%
MLVMF	43.58	37.86	30.01	23.95	19.67
NFMO (PA)	42.92	38.65	35.99	33.13	29.99

**Table 2:** Operational principles of the selected methods: Performances evaluation phase.

Method	Full Denomination	Year	Family	Principle
ASWM [35]	Adaptive Switching Weighted Median filter	2016	Median Filter-Based	The noise detection step compares the corrupted candidate to the local mean value to determine whether a pixel is “noise-free” or “noisy”. An adaptive weighted median filter replaces noisy pixels with weighted median values.
NAFSM [36]	Noise Adaptive Fuzzy Switching Median filter	2010	Median Filter-Based	A histogram determines the contaminated pixel. The filtering step processes the noisy pixel while maintaining the noise-free pixel components. Local maximums at the histogram ends help the algorithm find the erroneous pixel. A Boolean noisy mask identifies the noisy pixel. The noisy pixel receives 0, and the rest receive 1. The predicted correction term replaces a noisy pixel during filtering based on the mask’s notation.
MDBUTMF [37]	Modified Decision-Based Un-symmetric Trimmed Median Filter	2011	Median Filter-Based	If brightness levels fall between the contrasting gray values, the pixel element is unchanged. MDBUTMF regulates intermediate pixels.
ADWMF [38]	Adaptive Dynamically Weighted Median Filter	2017	Median Filter-Based	A weighted median filter with a simple impulse detector is used. Noise density determines window size. After detecting impulsive noise, the weighted median filter gives the noisy element zero weight in the next frame.
DBIDWI [39]	Adaptive Decision Based Inverse Distance Weighted Interpolation	2017	Interpolation-Based	Inverse distance-weighted interpolations replace noisy pixels. Based on nearby non-noisy pixels, this interpolation predicts damaged pixel values.
ADKIF [40]	Adaptive Decision Based Kriging Interpolation Filter	2018	Interpolation-Based	The algorithm processes only noisy pixels. A weighted interpolation replaces the faulty pixel. If a processing window has less than three non-noisy pixels, the window increases adaptively.
PDBF [41]	Probabilistic Decision Based Filter	2016	Probability and Statistics	Based on the anticipated noise density, the filter employs either TM (Trimmed Median) or PETM (Patch Else Trimmed Median), resulting in improved denoising performance.
APF [42]	Adaptive Probability Filter	2018	Probability and Statistics	In order to identify noise, the pixel is compared to its neighboring pixel to establish its appropriateness as an image element. To replace the processing pixel, the number of noise-free pixel components is computed and compared to the pixel’s estimated threshold value. If no noise-free components are discovered, the mean filter is used in their place.
BPDF [43]	Based-on-Pixel Density Filter	2018	Probability and Statistics	A range is selected between the two extremes values around a pixel. The algorithm searches for at least one pixel inside and beyond that range. Beyond the range, it is presumed that the pixel is noise-free. If so, the most repeated pixel intensity is searched for a second time. The median of the repeating pixel is substituted for the processing pixel.
ACCPPRA [44]	Adaptive Content based Closer Proximity Pixel Replacement Algorithm	2020	Heuristic Decision Tree	Calculate the Euclidean distance between the processed pixel and nearby non-corrupted pixels. The technique expands the window if the processing kernel has no non-noisy pixels. The median of pixels that occur more frequently in the current processing window replaces erroneous pixels based on Euclidean distance.
AWMF [45]	Adaptive Weighted Mean Filter	2014	Mean filter-Based	The method progressively increases the window size until the maximum and minimum values of two succeeding windows match. The current pixel may be noisy if its value matches the highest or lowest. The window weighted mean replaces the noise candidate.
ACBSA [46]	Adaptive Cardinal B-Spline Algorithm	2012	Cardinal B-Spline Analysis	This approach conducts a cardinal B-spline analysis and application for picture noise removal. To apply cardinal B-splines, one must study the cardinal B-many spline’s qualities. Here, cardinal B-splines’ approximation function and compact support are used.

**Table 3:** Performances of selected methods and performances of the proposed NFMO method at different noise densities (from 10% to 90%) for PSNR on the Lena standard image.

Noise in % 🡪	10%	30%	50%	70%	90%
ASWM	42.54	36.09	32.16	28.61	23.11
NAFSM	39.36	34.52	31.88	25.66	20.11
MDBUTMF	40.76	34.82	32.98	26.46	21.18
ADWMF	38.68	34.44	28.68	24.46	21.33
DBIDWI	43.01	38.50	35.68	32.44	28.45
ADKIF	44.48	40.02	35.85	32.48	27.11
PDBF	42.87	39.89	35.72	31.11	24.15
APF	43.55	40.77	36.14	32.64	25.53
BPDF	41.17	38.34	34.17	27.63	22.78
ACCPPRA	45.71	40.89	36.09	32.91	28.04
AWMF	39.37	36.94	34.57	31.38	25.97
ACBSA	41.90	37.10	33.80	29.80	26.60
NFMO (PA)	42.92	38.65	35.99	33.13	29.99
NFMO (PA)rank (out of 13)	5th	5th	3rd	1st	1st

**Table 4:** Performances of selected methods and performances of the proposed NFMO method at different noise densities (from 10% to 90%) for PSNR on the Boat standard image.

Noise in % 🡪	10%	30%	50%	70%	90%
MDBUTMF	32.11	29.67	26.15	21.71	18.81
DBIDWI	36.95	33.77	30.45	27.86	24.33
ADKIF	36.20	33.07	30.02	27.05	22.41
ACCPPRA	37.17	33.51	30.27	27.87	23.76
AWMF	34.31	31.35	29.10	26.45	21.75
ACBSA	35.90	31.22	28.33	24.72	22.19
NFMO (PA)	36.24	33.40	30.42	29.18	27.20
NFMO (PA)rank (out of 7)	3rd	3rd	2nd	1st	1st

**Table 5:** Performances of selected methods and performances of the proposed NFMO method at different noise densities (from 10% to 90%) for PSNR on the Baboon standard image.

Noise in % 🡪	10%	30%	50%	70%	90%
MDBUTMF	29.92	28.41	27.08	24.07	19.24
DBIDWI	31.15	29.79	26.98	24.74	22.12
ADKIF	31.88	29.64	26.90	24.80	22.01
ACCPPRA	32.41	29.82	26.99	24.72	22.28
AWMF	30.78	28.69	26.63	24.60	21.42
ACBSA	31.17	28.13	25.71	22.54	20.65
NFMO (PA)	32.12	29.77	27.17	25.90	24.42
NFMO (PA)rank (out of 7)	2nd	3rd	1st	1st	1st

**Table 6:** Performances of NFMO in terms of PSNR and NCD were applied to six medical images at different noise densities.

Noise in % 🡪	10%		30%		50%		70%		90%	
Metrics 🡪	NCD	PSNR	NCD	PSNR	NCD	PSNR	NCD	PSNR	NCD	PSNR
CI1	0.0462	43.35	0.0633	39.42	0.0800	37.13	0.0963	35.17	0.1248	31.94
CI2	0.1290	43.10	0.1442	39.08	0.1574	36.46	0.1672	34.71	0.1775	31.05
CI3	0.0048	39.75	0.0131	37.42	0.0208	35.20	0.0281	34.34	0.0371	33.45
CI4	0.0061	39.29	0.0165	37.58	0.0262	36.36	0.0350	35.76	0.0462	34.11
CI5	0.0396	42.25	0.0563	37.99	0.0710	35.67	0.0843	33.91	0.1031	31.33
CI6	0.0520	37.51	0.0738	33.46	0.0924	31.18	0.1071	29.78	0.1293	27.84

**Table 7:** FPGA resources and NFMO-designed architectures’ performance.

	LUT	FF	BRAM_18K	DSP48E	Cycles
**Solution 1**	12,023 (4%)	5099 (1%)	121 (7%)	16 (0.5%)	220,023,441
**Solution 2**	49,535 (18%)	44,656 (8%)	121 (7%)	612 (24%)	5,503,836
**Solution 3**	54,667 (20%)	49,776 (9%)	140 (8%)	613 (24%)	2,688,422

## Data Availability

Not applicable.
